# Mutations in *FIGLA* Associated With Premature Ovarian Insufficiency in a Chinese Population

**DOI:** 10.3389/fmed.2021.714306

**Published:** 2021-10-29

**Authors:** Libin Mei, Yanru Huang, Xiaoling Wu, Huang He, Ronghui Ye, Jinxiu Ma, XueMei He, Yuhua Shi, Ping Li

**Affiliations:** ^1^Department of Reproductive Medicine, Women and Children's Hospital, School of Medicine, Xiamen University, Xiamen, China; ^2^Xiamen Key Laboratory of Reproduction and Genetics, Women and Children's Hospital, School of Medicine, Xiamen University, Xiamen, China; ^3^School of Public Health, Xiamen University, Xiamen, China; ^4^Center for Reproductive Medicine, Cheeloo College of Medicine, Shandong University, Jinan, China; ^5^Shandong Provincial Clinical Medicine Research Center for Reproductive Health, Shandong University, Jinan, China

**Keywords:** *FIGLA*, zona pellicuda, chromatin immunoprecipitation, transcriptional, premature ovarian insufficiency

## Abstract

**Objective:** Premature ovarian insufficiency (POI) is one of the most common reproductive endocrinological causes of infertility in women of child-bearing age. The purpose of this study was to identify *FIGLA* gene mutations in Chinese patients with POI and to investigate the underlying mechanism.

**Methods:** A total of 113 patients with idiopathic POI and 100 healthy controls were recruited for the analysis of *FIGLA* variants. Based on the identification of common mutations in the *FIGLA*, wild-type and mutant plasmids were constructed and transfected into HEK293 cells. Luciferase reporter genes were used to determine the effect of wild-type and mutant *FIGLA* genotypes on the transcriptional activity of its downstream targets, the zona pellucida glycoprotein genes *ZP1, ZP2*, and *ZP3*. Chromatin immunoprecipitation was used to determine the level of binding between wild-type and mutant *FIGLA* with the *ZP1, ZP2*, and *ZP3* promoters.

**Results:** Three different *FIGLA* mutations were identified in four patients with POI. Two patients carried the mutation c.11C>A (p.A4E), and the other two patients, respectively, carried the mutations c.625G>A (p.V209I) and c.84C>A (p.D28E). The luciferase reporter assay indicated that *ZP1, ZP2*, and *ZP3* transcriptional activities were significantly reduced in individuals with *FIGLA* mutations. Chromatin immunoprecipitation indicated that the *FIGLA* mutation significantly decreased binding with the *ZP1, ZP2*, and *ZP3* promoters.

**Conclusion:** *FIGLA* mutation affects gene transcriptional regulation of its downstream target genes *ZP1, ZP2*, and *ZP3*, highlighting a new candidate genetic factor that causes POI. Our study demonstrates that *FIGLA* has a regulatory effect on reproduction-specific genes, thereby providing a basis for elucidating the specific regulatory mechanism of *FIGLA* in germ cell growth and development.

## Introduction

Premature ovarian insufficiency (POI) refers to non-physiological amenorrhea due to ovarian failure in women before the age of 40. POI is accompanied by increased serum gonadotropin levels and decreased estrogen levels, as well as typical clinical manifestations of hot flashes, excessive sweating, facial flushing, and decreased libido, among other symptoms (1, 2). The incidence of POI is about 3.9% (3), and is a major factor leading to female infertility. The etiology of POI is complex, which includes hereditary, autoimmune, iatrogenic, infection, and environmental factors, and its pathogenic mechanism remains unclear. Of these, hereditary factors such as chromosomal abnormalities and gene mutations (4) have long been regarded as the major pathogenic factors of POI. Primordial follicle formation is an important signal that establishes reproductive potential in women and is also a key factor in determining whether POI or premature menopause develops. Several genes have been identified to play a potential role in the formation and development, including *NOBOX, FIGLA, SOHLH1, LHX8, FOXO3*, and other follicular development-specific transcription factors (5–8).

Among these, the folliculogenesis-specific basic helix-loop-helix (bHLH) transcription factor gene (*FIGLA*; MIM 608697) is one of the earliest expressed germ cell-specific transcription factors. *FIGLA* is located on human chromosome 2p13.3 and encodes a bHLH protein, a highly conserved family of transcriptional regulatory factors that bind cis-acting elements in the promoter region of genes (9). *FIGLA* plays an important role in regulating the development of primordial follicles, the expression of zona pellucida genes, and the formation of the zona pellucida (10). In both mice and humans, *FIGLA* is expressed at an early stage of development in the embryonic gonads, where it targets and binds zona pellucida genes (*ZP1, ZP2*, and *ZP3*), together with TCF3 protein, thereby regulating the expression of the zona pellucida genes and other oocyte factors (11, 12). Mutation of *FIGLA* may affect the transcription of zona pellucida genes and cause ovarian dysfunction. Male mice are unaffected by *Figla* knockout, whereas female mice lacking *Figla* do not express *Zp1, Zp2*, or *Zp3*, and thus cannot form the zona pellucida. Although the gonads of *Figla*-knockout mice develop normally in the embryonic stage, they cannot form primordial follicles after birth, resulting in the loss of many oocytes and consequently sterility (13). However, the role of *FIGLA* in human POI remains unclear.

In this study, we used Sanger sequencing to screen the *FIGLA* gene mutations in 113 Chinese patients with POI. Three heterozygous missense mutations were found in four POI patients. Western blotting, luciferase reporter gene assays, chromatin immunoprecipitation (ChIP), and other molecular biology tools were then used to investigate the effect of these *FIGLA* mutations on the transcription of its downstream targets *ZP1, ZP2*, and *ZP3*. These findings can provide new insight into the underlying pathogenic mechanism of POI, clarify the roles of *FIGLA* in germ cell development and function, and indicate new treatment targets.

## Results

### Genetic Analysis and Functional Prediction of *FIGLA* Mutations

**Figure 1:**
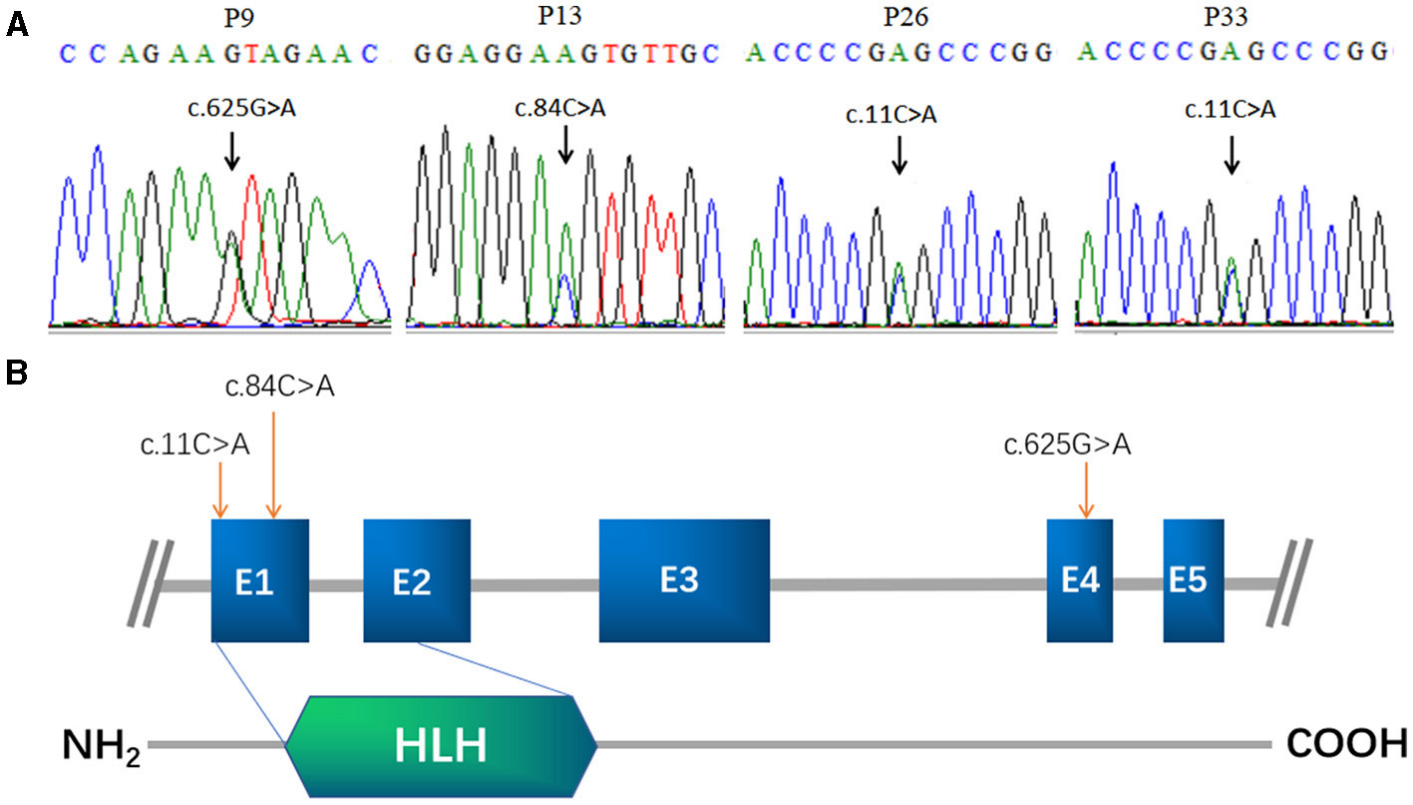
*FIGLA* mutation identification in POI patients. Sequencing showing the c.625G>A mutation in patient 9, the c.84C>A mutation in patient 13 **(A)**, and the c.11C>A mutation in patient 26 and patient 33 **(B)**. The human *FIGLA* gene is schematically drawn with five known exons (blue boxes); the c.84C>A and c.11C>A mutations are located in the *FLGLA* HLH domain (green rhombus).

Among the 113 Chinese POI subjects, we identified three missense mutations in the *FIGLA* gene in four patients (Figure 1A): c.11C>A (p.A4E), c.84C>A (p.D28E), and c.625G>A (p.V209I), which have been already reported in the online dbSNP database (rs71647803, rs373561603, and rs186548772, respectively). All these variations were classified as likely pathogenic according to ACMG criteria. One POI patient (P9) carried a missense mutation c.625G>A, which caused a change in the amino acid at position 209 of the encoded protein from valine to isoleucine (p.V209I). The allele frequency in the ExAC database was 0.003. This variant was predicted to be deleterious by MutationTaster and M-CAP, and was not present in any of the healthy controls. P9 presented with irregular amenorrhea at 34 years of age. Menarche occurred when she was 16 years old. Follicle-stimulating hormone (FSH) measurements were taken twice, with values of 52.8 and 60.4 IU/L, respectively. Transvaginal ultrasound examination revealed bilateral ovarian atrophy and the absence of ovarian follicles. Sanger sequencing confirmed that the c.625G>A mutation of P9 was inherited from her father, who has no evident somatic anomalies.

Another patient (P13) had a missense mutation, c.84C>A (p.D28E), in FIGLA, which is located in the highly conserved HLH domain and caused a change in the amino acid at position 28 from aspartic acid to glutamic acid. P13 was 25 years old. The allele frequency in the ExAC database was 0.004, but not present in any of the control subjects. The use of online pathogenesis prediction programs for this mutation resulted in “probably deleterious” by MutationTaster and “probably damaging” by M-CAP. Transvaginal ultrasound examination revealed that the size of the uterus was reduced (43 × 32 × 25 mm, whereas the normal size is 60 × 50 × 40 mm), the endometrium was thin, the left ovary was small (11 × 9 × 6 mm, whereas the normal size is 40 × 30 × 10 mm), ovarian follicles were absent, and the right ovary was not found (details are provided in Table 1). The patient inherited the mutation from her father, who is healthy and had no relevant fertility-related abnormalities.

**Table 1:** Phenotypes of women with POI carrying *FIGLA* mutations.

**Patient**	Age^*^	**Karyotype**	**FSH(IU/L)**	**LH(IU/L)**	**E_**2**_ (pg/ml)**	**AMH(ng/ml)**	**Sequence variation**	**Protein position**	**Predictions MutTas/M-CAP**	**Classification ACMG**	**Transabdominal ultrasonography**
P9	34	46,XX	52.8^a^; 60.4^b^	21.7^a^; 12.3^b^	27.2^a^; 22.1^b^	0.14	c.625G>A	p.V209I	Probably deleterious/probably damaging	Likely pathogenic	Atrophic uterus and ovaries not visualized
P13	25	46,XX	65.3^a^; 71.5^b^	39.6^a^; 43.6^b^	31.5^a^; 27.8^b^	0.10	c.84C>A	p.D28E	Probably deleterious/probably damaging	Likely pathogenic	A small left ovary (11 ×9 ×6 mm) devoid of follicles. The right ovary was not visualized
P26	27	46,XX	85.0^a^; 100.6^b^	43.5^a^; 36.6^b^	51.8^a^; 40.8^b^	0.09	c.11C>A	p.A4E	Probably harmless/probably damaging	Likely pathogenic	Atrophic uterus and ovaries not visualized
P33	22	46,XX	77.4a; 88.7b	40.6^a^; 52.3^b^	43.7^a^; 29.6^b^	0.14	c.11C>A	p.A4E	Probably harmless/probably damaging	Likely pathogenic	Atrophic uterus and ovaries not visualized

*FIGLA denotes factor in the germline alpha; FSH denotes follicle-stimulating hormone; LH denotes luteinizing hormone; E2 denotes estradiol*.

* *Age of onset*.

a *Serum levels; initial value*.

b *Serum levels; repeat value*.

The other two POI patients (P26 and P33) harbored the missense mutation c.11C>A in the highly conserved HLH domain (Figure 1B), causing a change in the amino acid at position 4 of the encoded protein from alanine to glutamic acid (p.A4E). This variant was not present in the control cases, and was predicted to be “probably harmless” and “probably damaging” using *in silico* MutationTaster and M-CAP programs, respectively. Each of these patients inherited the mutation from their father. P26 and P33 were 27 and 22 years of age, respectively. Both patients had secondary amenorrhea and infertility. Transvaginal ultrasound examination revealed bilateral ovarian atrophy and the absence of ovarian follicles.

### Successful Transfection of HEK293 Cells With Wild-Type and Mutant Plasmids

**Figure 2:**
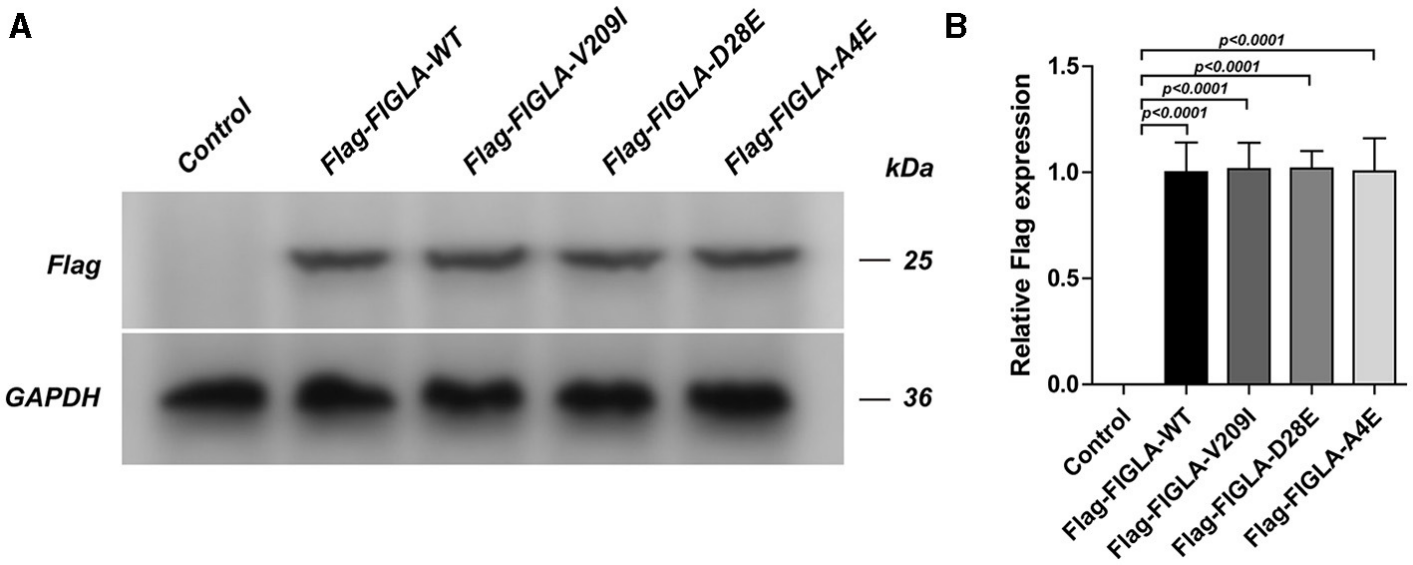
HEK293 cells transfected with Flag-FIGLA-WT, Flag-FIGLA-V209I, Flag-FIGLA-D28E, or Flag-FIGLA-A4E. The transfection efficiency was detected by western blot assay **(A,B)**. Results were mean ± SD for three individual experiments which, for each condition, were performed in triplicate.

The HEK293 cells were transfected with corresponding plasmids harboring wild-type *FIGLA* or the three mutations identified in the POI patients. Western blotting confirmed the good transfection efficiency of the wild-type and mutant plasmids (Figure 2).

### *FIGLA* Mutants Exhibited Significantly Reduced Transcriptional Activity of *ZP1, ZP2*, and *ZP3*

**Figure 3:**
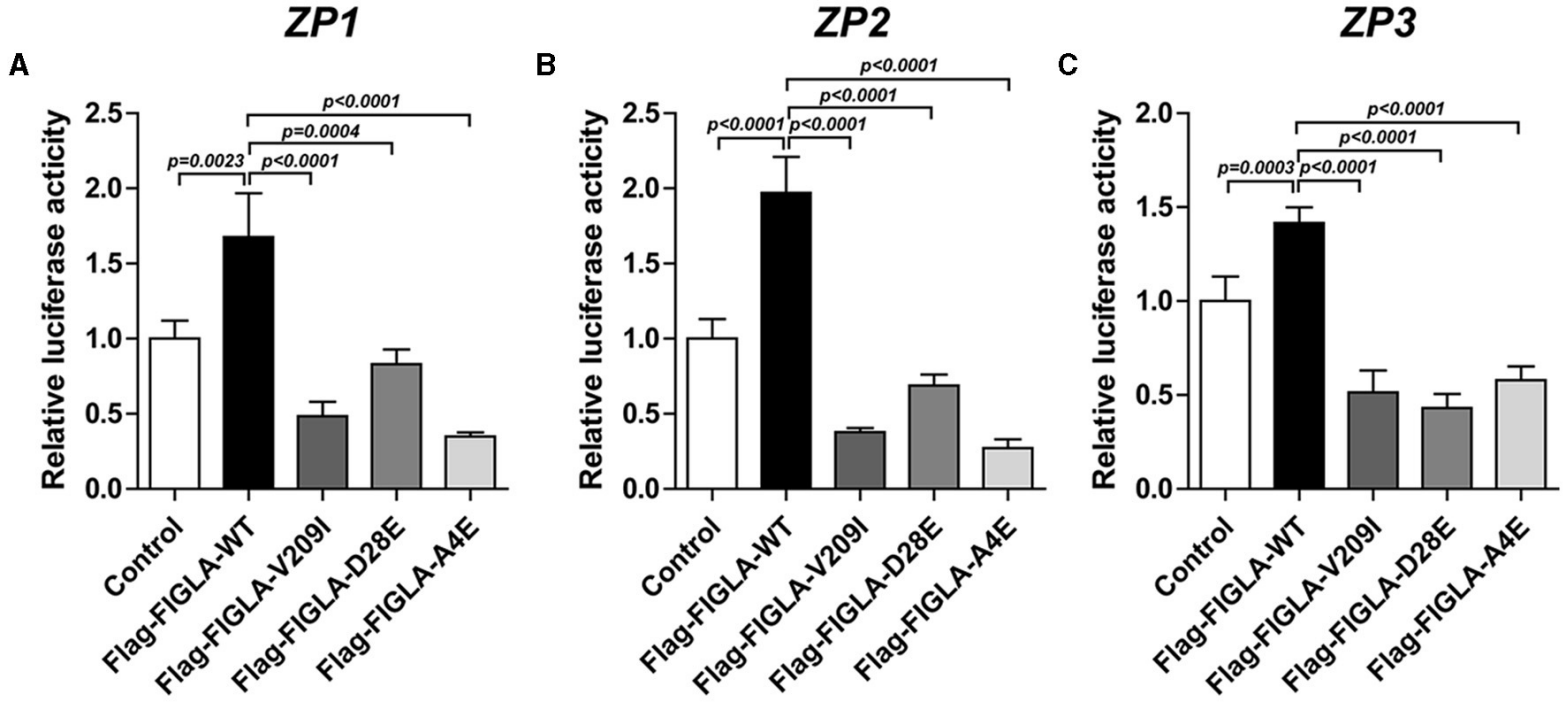
*FIGLA* mutation impairs the transcriptional activity of *ZP1, ZP2*, and *ZP3*. HEK293 cells were transfected with Flag-FIGLA-WT, Flag-FIGLA-V209I, Flag-FIGLA-D28E, or Flag-FIGLA-A4E. The transcriptional activity of *ZP1* **(A)**, *ZP2* **(B)**, and *ZP3* **(C)** promoter fragments was detected by a luciferase reporter assay. Results were mean ± SD for three individual experiments which, for each condition, were performed in triplicate.

The effects of wild-type and mutant *FIGLA* on *ZP1, ZP2*, and *ZP3* transcriptional activity were confirmed through luciferase reporter gene reporter assays. The cells harboring the *FIGLA* mutant plasmids Flag-FIGLA-V209I (*p* < 0.0001), Flag-FIGLA-D28E (*p* < 0.0004), and Flag-FIGLA-A4E (*p* < 0.0001) had significantly reduced transcriptional activities of *ZP1* compared with those of the wild-type (Figure 3A). The cells harboring the *FIGLA* mutant plasmids Flag-FIGLA-V209I(*p* < 0.0001), Flag-FIGLA-D28E(*p* < 0.0001), and Flag-FIGLA-A4E (*p* < 0.0001) had significantly reduced transcriptional activities of *ZP2* compared with those of the wild-type (Figure 3B). The cells harboring the *FIGLA* mutant plasmids Flag-FIGLA-V209I (*p* < 0.0001), Flag-FIGLA-D28E (*p* < 0.0001), and Flag-FIGLA-A4E (*p* < 0.0001) had significantly reduced transcriptional activities of *ZP3* compared with those of the wild-type (Figure 3C).

### *FIGLA* Mutants Exhibited Significantly Reduced Binding to the *ZP1, ZP2*, and *ZP3* Promoters

**Figure 4:**
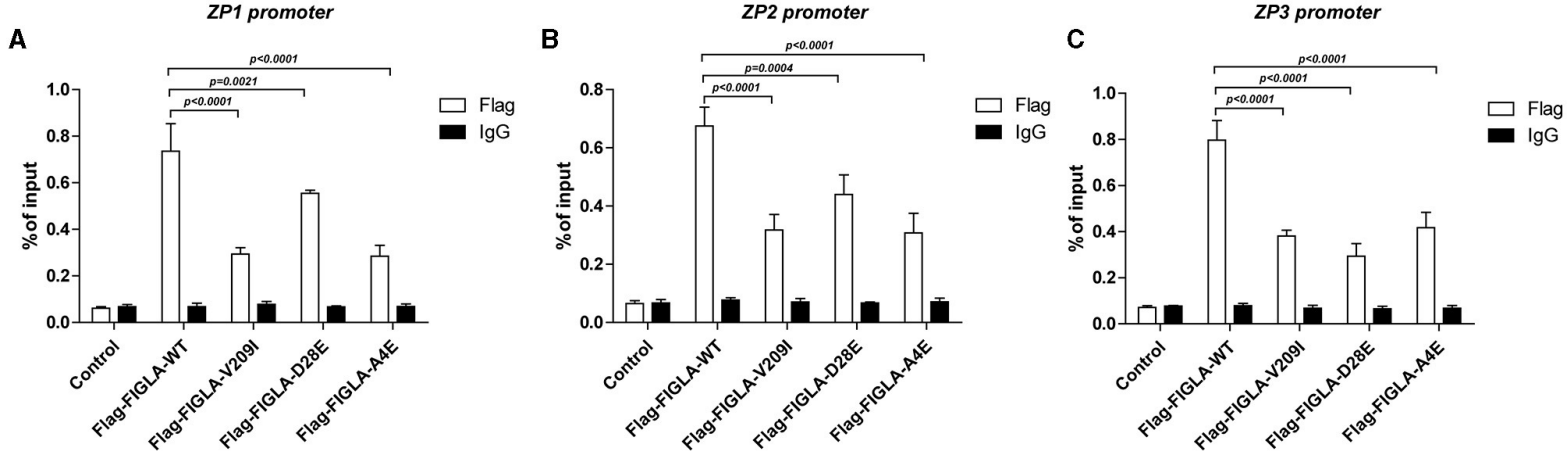
*FIGLA* mutation impairs binding of *FIGLA* to the promoter regions of *ZP1, ZP2*, and *ZP3*. HEK293 cells were transfected with Flag-FIGLA-WT, Flag-FIGLA-V209I, Flag-FIGLA-D28E, or Flag-FIGLA-A4E. Interaction of *FIGLA* with the promoter region of *ZP1* **(A)**, *ZP2* **(B)**, and *ZP3* **(C)** was detected by chromatin immunoprecipitation assays. Results were mean ± SD for three individual experiments which, for each condition, were performed in triplicate.

The differences in promoter binding of wild-type and mutant *FIGLA* with the *ZP1, ZP2*, and *ZP3* promoters were determined using ChIP experiments. The plasmids harboring the *FIGLA* mutants Flag-FIGLA-V209I (*p* < 0.0001), Flag-FIGLA-D28E (*p* < 0.0021), and Flag-FIGLA-A4E (*p* < 0.0001) showed significantly reduced binding to the *ZP1* promoters compared with the wild-type (Figure 4A). The plasmids harboring the *FIGLA* mutants Flag-FIGLA-V209I (*p* < 0.0001), Flag-FIGLA-D28E (*p* = 0.0004), and Flag-FIGLA-A4E (*p* < 0.0001) showed significantly reduced binding to the *ZP2* promoters compared with the wild-type (Figure 4B). The plasmids harboring the *FIGLA* mutants Flag-FIGLA-V209I (*p* < 0.0001), Flag-FIGLA-D28E (*p* < 0.0001), and Flag-FIGLA-A4E (*p* < 0.0001) showed significantly reduced binding to the *ZP3* promoters compared with the wild-type (Figure 4C).

## Discussion

Premature ovarian insufficiency is a disease of the gynecological endocrine system with high clinical and genetic heterogeneity. POI can be caused by mutations in genes involved in the formation, growth, and development of follicles; genes involved in the synthesis and action of sex hormones; or genes involved in meiosis; and DNA damage repair (4). In the present study, we screened 113 Han Chinese patients with POI through Sanger sequencing and found three heterozygous missense mutations in *FIGLA* associated with follicle formation. Two of the patients carried the mutation c.11C>A (p.A4E), and the other two patients carried the missense mutations c.625G>A (p.V209I) and c.84C>A (p.D28E), respectively. These findings will help researchers and clinicians better understand the genetic etiology of POI and can support genetic counseling of patients.

Previous studies have shown that mutation and abnormal expression of the human *FIGLA* gene can cause developmental disorders in female germ cells, affecting primary follicle recruitment, and leading to secondary amenorrhea, infertility, POI, and other disorders (12, 14, 15). Zhao et al. (6) screened 100 Chinese patients with POI and found two patients with secondary amenorrhea who carried the missense mutation c.11C->A (p.A4E), which was consistent with the mutation site found in the present study. This mutation is located in the highly conserved HLH domain; the other two mutations were deletions found at c.15-36del (p.G6fsX66) and c.419-421del ACA (p.140delN). The G6fsX66 frameshift mutation leads to haploinsufficiency and is associated with POI, and functional analysis indicated that the abnormal FIGLA function could be due to dominant negative interference of the 140 delN protein on the binding of WT FIGLA to TCF3. Tosh et al. (16) detected seven different mutations in *FIGLA* in a screen of 219 Indian patients with idiopathic POI, indicating that the *FIGLA* gene is a potential genetic risk factor for POI. Yuan et al. (17) revealed the recessive genetic inheritance pattern of *FIGLA* mutations, in which the biallelic *FIGLA* mutation c.2T>C led to the development of POI in a family with primary amenorrhea. The four patients with identified *FIGLA* mutations in the present study all carried heterozygous mutations and exhibited secondary amenorrhea, indicating that symptoms of POI caused by homozygous mutations may be more severe than those caused by haploinsufficiency of the *FIGLA* gene. However, with the current data we cannot exclude the possibility that the new protein created by the heterozygous variants adversely impairs WT FIGLA function *via* dominant negative interaction or haploinsufficiency.

*FIGLA* is a transcriptional regulator of the key downstream target genes *ZP1, ZP2*, and *ZP3* during early follicular development. Functional studies have found that *FIGLA* binds to the E-box upstream of the *ZP* genes to regulate their expression, thereby regulating the formation of the zona pellicuda (12). FIGLA can mediate *ZP* transcription under normal circumstances, but mutations may affect their transcriptional activity, resulting in ovarian dysfunction. Therefore, we examined whether the *FIGLA* mutations affected *ZP* transcriptional activity. ChIP experiments showed that the cells harboring plasmids with the *FIGLA* mutants Flag-FIGLA-V209I, Flag-FIGLA-D28E, and Flag-FIGLA-A4E exhibited significantly decreased binding with the *ZP1, ZP2*, and *ZP3* promoters. Luciferase reporter assays further showed that these *FIGLA* mutants led to significantly decreased *ZP1, ZP2*, and *ZP3* transcriptional activity. Liang et al. (18) found that *ZP1, ZP2*, and *ZP3* are not expressed or are abnormally expressed in female mice with FIGLA deficiency, and these mice lacked primordial follicles and were infertile. Together, these results indicate that *FIGLA* mutations affect the regulation and normal transcription of *ZP* genes, which may disrupt the normal formation of the zona pellicuda, cause disorders of oocyte maturation, and lead to POI and infertility.

In summary, we identified three plausible POI-causal mutations of *FIGLA* in Chinese POI patients, which affected the transcriptional regulation of downstream target genes (*ZP1, ZP2*, and *ZP3*). The present study shows that *FIGLA* plays a regulatory role in reproduction-specific genes, thereby providing a basis for further understanding of the specific regulatory mechanisms of *FIGLA* in germ cell growth and development. Elucidating the genetic etiology and molecular mechanism of POI will not only improve understanding of ovarian physiology but will also provide a theoretical basis for genetic counseling and family planning of high-risk clinical populations. Identification of new genes and pathways involved in the pathogenesis of POI offer future targets for drug therapy, thereby establishing a basis for developing an actual cure for POI.

## Materials and Methods

### Participants

This study included 113 patients with POI who were recruited from the Department of Reproductive Medicine, Women and Children's Hospital, Xiamen University (Xiamen, China). All participants were clinically evaluated and a complete medical history was obtained, including menstrual history; age at menopause; serum FSH, luteinizing hormone, thyroid-stimulating hormone, and anti-Müllerian hormone levels; and history of any autoimmune diseases or psychiatric disorders. The diagnostic criteria for POI are at least 6 months of amenorrhea before the age of 40 years and high serum FSH levels (≥25 IU/L). A detailed inquiry of medical history, along with the results of physical, gynecological, and ultrasound examinations were used to exclude iatrogenic, immunological, and infection-related factors as well as chromosomal abnormalities. In addition, 100 healthy women between the ages of 23 and 40 years with normal ovarian function and no history of infertility were selected as healthy controls for comparison. All patients and healthy individuals signed letters of informed consent. The study was conducted after approval by the Institutional Review Board of Women and Children's Hospital, Xiamen University.

### Molecular Genetic Analyses

Genomic DNA was extracted from peripheral blood leukocytes of the patients using the QIAamp blood kit (QIAGEN, Hilden, Germany) following the protocol of the manufacturer. The entire coding region and the intron–exon boundaries of the FIGLA gene were amplified by the polymerase chain reaction (PCR). The PCR primers were designed using Primer5 software (Primer Biosoft International, Vancouver, Canada). The PCR products were confirmed by polyacrylamide gel electrophoresis (PAGE) and directly sequenced with a Taq-Dideoxy terminator cycle sequencing kit using an automated ABI 3730 sequencer (Applied Biosystems, Carlsbad, USA). The sequences were aligned and analyzed using the DNASTAR (Madison, USA) software. The genomic sequence of the FIGLA gene (NM_001004311.3) was used as the reference sequence. The pathogenicity of the variants identified was classified in accordance with the guidelines of the American College of Medical Genetics and Genomics (ACMG). Furthermore, online tools such as MutationTaster and M-CAP were applied to evaluate the pathogenicity of the detected SNP variants.

### Construction of Wild-Type and Mutant *FIGLA* Plasmids

Primers specific to the *FIGLA* sequence were designed as follows: wild-type plasmid Flag-FIGLA-WT forward 5′-CTAGGATCCTATCCTTAAAGGTGCGACTG-3′, reverse 5′-ATTGAATTCTCTTCATTCTTCAAGCCGAA-3′; V209I mutation forward 5′-CCAGAAATCGAACTGCTGAGTCACAGAC-3′, reverse 5′-AGTTCGATTTCTGGGAATCTATCCAGAC-3′; D28E mutation forward 5′-TGGAGGAGGTGTTGCGGGAGCAGTT-3′, reverse 5′-AACACCTCCTCCAGCACCTCGGCTT-3′; and A4E mutation forward 5′-CCCGAGCCCGGCGTCCTAGAT-3′, reverse 5′-TAGGACGCCGGGCTCGGGGTCCAT-3′. The mutant plasmids Flag-FIGLA-V209I, Flag-FIGLA-D28E, and Flag-FIGLA-A4E were constructed using the QuickMutation Plus gene-directed mutagenesis kit per the manufacturer's instructions. HEK293 cells were divided into a control group, Flag-FIGLA-WT group, Flag-FIGLA-V209I group, Flag-FIGLA-D28E group, and Flag-FIGLA-A4E group. After transfection of the corresponding plasmid, the transfection efficiency of each group was verified by western blotting.

### Western Blot Analysis

Total protein was collected and extracted from the cells using radioimmunoprecipitation buffer and the protein concentration was determined using the bicinchoninic acid method. Protein samples (30 μg) were added to each well of a sodium dodecyl sulfate–polyacrylamide gel electrophoresis gel, and the electrophoresis program was set to 80 V for 30 min and 120 V for 1 h. Wet transfer to polyvinylidene fluoride (PVDF) membrane was performed at constant pressure at 100 V for 1.5 h. The diluted primary antibody was added and incubated overnight at 4°C. The membrane was washed three times with Tris-buffered saline with Tween (TBST) for 10 min each, and then incubated with the diluted secondary antibody corresponding to the primary antibody for 1 h at room temperature. The membrane was washed again three times with TBST for 10 min each. Equal volumes of chemiluminescence reagents were mixed and applied to the membrane. The Tanon 6600 luminescence imaging system was used for imaging. Image Pro Plus 6.0 software was used to analyze the optical density of the bands to quantify protein expression, and the relative protein expression level was calculated as the grayscale value of the target protein/grayscale value of the internal reference protein.

### Luciferase Reporter Assay

Primers specific to the promoter sequences of the *FIGLA* target genes (*ZP1, ZP2*, and *ZP3*) were designed. RNA was extracted and reverse-transcribed into cDNA. Polymerase chain reaction (PCR) was then used to amplify preselected fragments of the target genes, and the PCR products were recovered and purified by agarose gel electrophoresis. The pGL3-REPORT plasmid and the purified *ZP* fragments (*ZP1, ZP2*, and *ZP3*) were digested, recovered, and ligated with T4 ligase to obtain the luciferase reporter gene, which was then amplified and transfected into HEK293 cells. After transfection with wild-type or mutant plasmids, the cells were transfected with Luc-ZP1, Luc-ZP2, and Luc-ZP3 reporter gene plasmid vectors. After 24 h, 100 μL of the reporter gene cell lysate was added to each well of a 96-well plate. After complete lysis, the lysate was centrifuged at 10,000–15,000 g for 3–5 min, the supernatant was collected, and luciferase activity was measured using a microplate reader.

### ChIP Experiments

The cells were incubated with formaldehyde at a final concentration of 1% for 10 min at 37°C to crosslink proteins and DNA. Crosslinking was terminated by treating the cells with glycine at a final concentration of 0.125 mol/L for 5 min at room temperature. The cells were collected and disrupted using a VCX750 sonicator (SONICS, USA) at an amplitude of 25%, pulse on for 4.5 s, and pulse off for 9 s for a total of 14 cycles. The cell lysate was centrifuged at 10,000 × *g* for 10 min at 4°C, insoluble material was removed, and the supernatant was collected. The specific antibody protein A agarose/salmon sperm DNA was used to bind to DNA-binding proteins, and the complexes were separated by precipitation. DNA was released and the proteins were digested by reverse crosslinking. The *ZP1, ZP2*, and *ZP3* promoter fragments were detected by PCR as described above.

### Statistical Analysis

All data are expressed as mean ± standard deviation. One-way analysis of variance and Tukey's *post-hoc* test were used to compare differences among groups. Differences with *p* < 0.05 were considered statistically significant.

## Data Availability Statement

The datasets presented in this study can be found in online repositories. The names of the repository/repositories and accession numbers can be found here: https://www.ncbi.nlm.nih.gov/snp/ rs71647803, rs373561603, rs186548772.

## Ethics Statement

The study was conducted after approval by the Institutional Review Board of Women and Children's Hospital, Xiamen University. The patients/participants provided their written informed consent to participate in this study. Written informed consent was obtained from the individual(s) for the publication of any potentially identifiable images or data included in this article.

## Author Contributions

LM and YH carried out research design, experiments, analyzed data, explained the results, and drafted manuscripts. XW and HH participated in the design and interpretation of the research results. XH was responsible for ultrasound examination and interpretation of the results. RY and JM were involved in the experiment. YS and PL participated in supervision, revising the manuscript, and its critical review. All authors contributed to the article and approved the submitted version.

## Funding

This study was supported by the National Natural Science Foundation of China (Grant no. 31801044), the Young and Middle-aged Personnel Training of Fujian Province (Grant no. 2020GGB064), the medical and Health Research Guidance Plan of Xiamen (Grant no. 3502Z20209195), and the Medical Science Research Foundation of Bethune (Grant no. QL002DS).

## Conflict of Interest

The authors declare that the research was conducted in the absence of any commercial or financial relationships that could be construed as a potential conflict of interest.

## Publisher's Note

All claims expressed in this article are solely those of the authors and do not necessarily represent those of their affiliated organizations, or those of the publisher, the editors and the reviewers. Any product that may be evaluated in this article, or claim that may be made by its manufacturer, is not guaranteed or endorsed by the publisher.
