# Corrigendum: Mutations in *FIGLA* associated with premature ovarian insufficiency in a Chinese population

**DOI:** 10.3389/fmed.2022.946150

**Published:** 2022-09-06

**Authors:** Libin Mei, Yanru Huang, Xiaoling Wu, Huang He, Ronghui Ye, Jinxiu Ma, XueMei He, Yuhua Shi, Ping Li

**Affiliations:** ^1^Department of Reproductive Medicine, Women and Children's Hospital, School of Medicine, Xiamen University, Xiamen, China; ^2^Xiamen Key Laboratory of Reproduction and Genetics, Women and Children's Hospital, School of Medicine, Xiamen University, Xiamen, China; ^3^School of Public Health, Xiamen University, Xiamen, China; ^4^Center for Reproductive Medicine, Cheeloo College of Medicine, Shandong University, Jinan, China; ^5^Shandong Provincial Clinical Medicine Research Center for Reproductive Health, Shandong University, Jinan, China

**Keywords:** *FIGLA*, zona pellicuda, chromatin immunoprecipitation, transcriptional, premature ovarian insufficiency

In the original article, there was an error in the sentence “In the present study, we screened 113 Han Chinese patients with POI through whole-exome sequencing and found three heterozygous missense mutations in FIGLA associated with follicle formation.”

The correction has been made to the **DISCUSSION** section, and the corrected sentence appears below:

“In the present study, we screened 113 Han Chinese patients with POI through Sanger sequencing and found three heterozygous missense mutations in FIGLA associated with follicle formation.”

In the original article, there was an error in the sentence “This study included 100 patients with POI who were recruited from the Department of Reproductive Medicine, Women and Children's Hospital, Xiamen University (Xiamen, China).”

The correction has been made to the **MATERIALS AND METHODS** section, “Participants” sub section and the corrected sentence appears below:

“This study included 113 patients with POI who were recruited from the Department of Reproductive Medicine, Women and Children's Hospital, Xiamen University (Xiamen, China).”

The authors apologize for this error and state that this does not change the scientific conclusions of the article in any way. The original article has been updated.

## Publisher's note

All claims expressed in this article are solely those of the authors and do not necessarily represent those of their affiliated organizations, or those of the publisher, the editors and the reviewers. Any product that may be evaluated in this article, or claim that may be made by its manufacturer, is not guaranteed or endorsed by the publisher.

