# Supplementary figures and images for: Mutations in FIGLA Associated With Premature Ovarian Insufficiency in a Chinese Population
Source: Front Med (Lausanne). 2021 Oct 29;8:714306. doi: 10.3389/fmed.2021.714306 (PMC8585841; doi:10.3389/fmed.2021.714306)

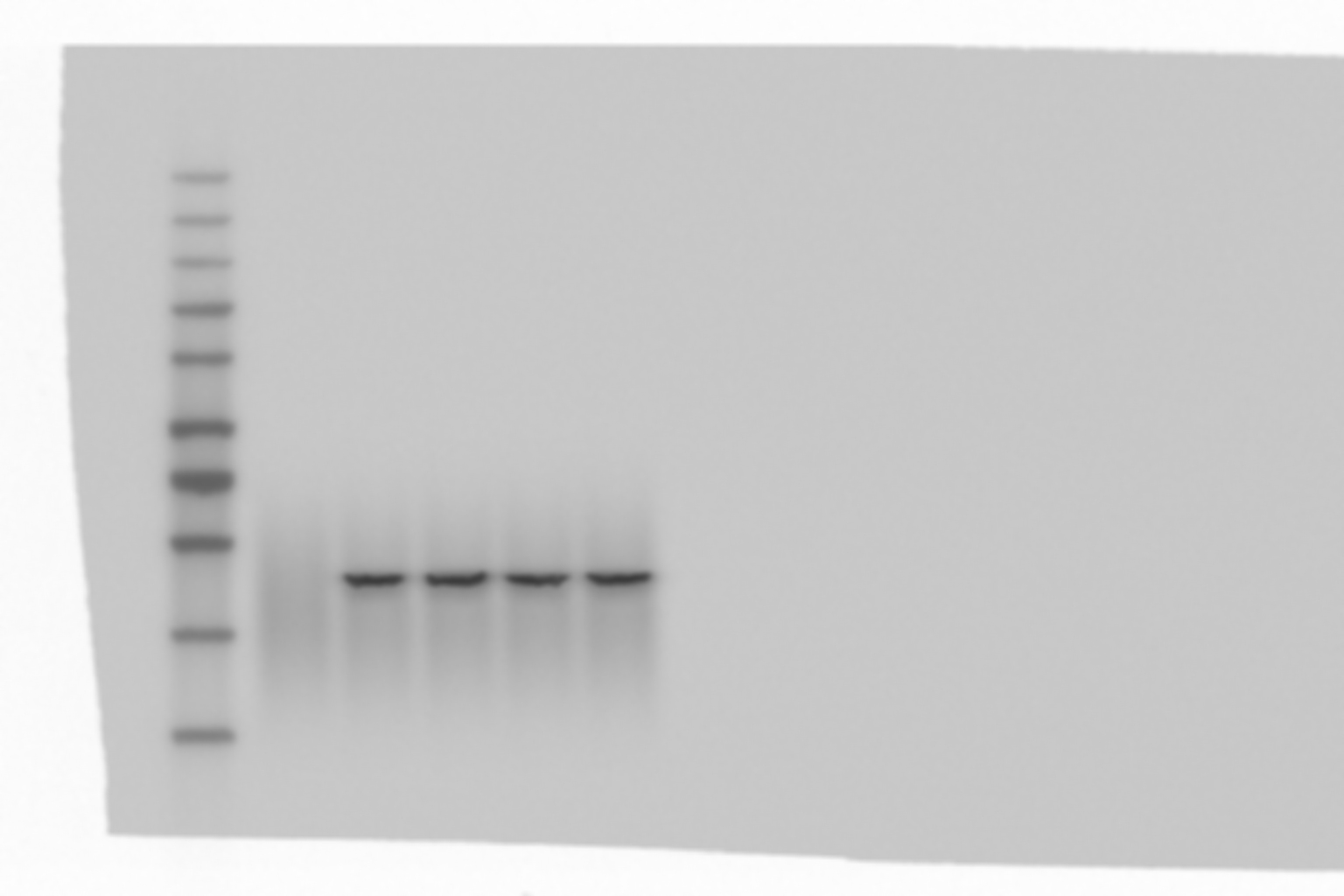

Supplement: Supplementary file 1 [file Image_1.JPEG]

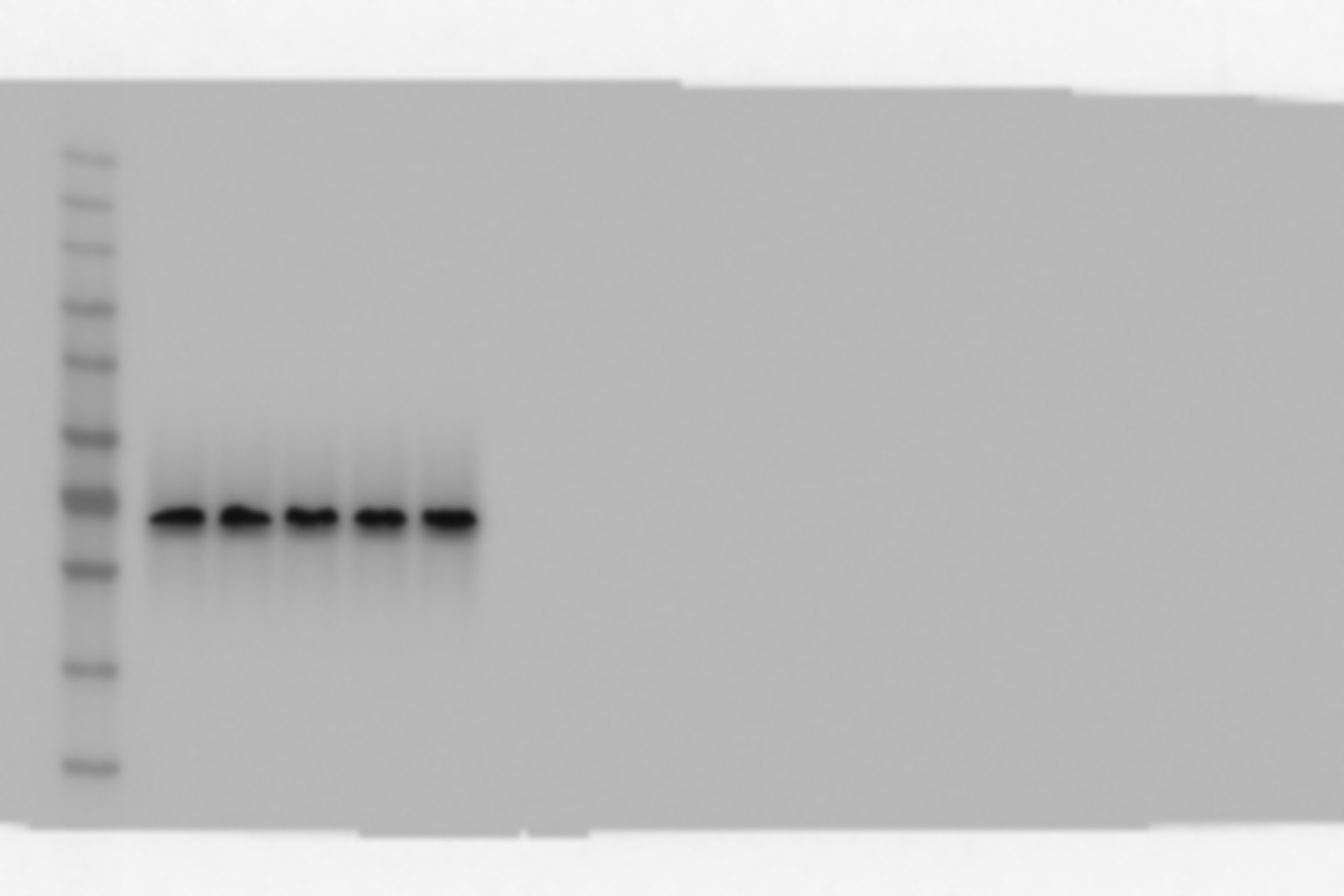

Supplement: Supplementary file 2 [file Image_2.JPEG]
